# A new function for the xanthophyll zeaxanthin: glueing chlorophyll biosynthesis to thylakoid protein assembly

**DOI:** 10.1042/BCJ20200803

**Published:** 2021-01-08

**Authors:** Roberto Bassi

**Affiliations:** Department of Biotechnology, University of Verona, Strada Le Grazie, 37134 Verona, Italy

## Abstract

Xanthophylls are coloured isoprenoid metabolites synthesized in many organisms with a variety of functions from the attraction of animals for impollination to absorption of light energy for photosynthesis to photoprotection against photooxidative stress. The finding by Proctor and co-workers makes a new addition to the last type of functions by showing that zeaxanthin is instrumental in coordinating chlorophyll biosynthesis with the insertion of pigment-binding proteins into the photosynthetic membrane by glueing the protein components catalyzing these functions into a supercomplex and regulating its activity.

Commentary on: Xanthophyll carotenoids stabilise the association of cyanobacterial chlorophyll synthase with the LHC-like protein HliD

By Matthew S. Proctor and co-workers

## 

The early definition of carotenoids as ‘accessory pigments’ in photosynthesis is misleading. These isoprenoid pigments are essential for both light harvesting and photoprotection. Indeed, chlorophylls, essential components for both light harvesting and charge separation reactions in all photosynthetic systems, have a major problem with oxygen: their triplet states react with O_2_ to yield singlet oxygen, a ROS (reactive oxygen species) which causes photodamage and photoinhibition. Chlorophyll, however, is the most abundant pigment on Earth, reaching ∼0.1 M in thylakoids. How each and all these innumerable potentially harmful molecules can possibly be prevented from producing ROS and destroying the photosynthetic systems they are aimed to serve is a rich chapter of biological studies. Among other examples is the peculiar folding mechanism of light-harvesting complex (LHC) pigment-proteins, binding most of the chlorophyll on earth: these chlorophyll–protein complexes can only fold by incorporating a set of xanthophylls (oxygenated carotenoids) [1] so that each of them comes into close contact with 3–4 chlorophylls [2]. Xanthophylls quench Chl triplets and/or prevent their formation by down-regulating the level of Chl singlet excited states [3], a function triggered whenever light intensity exceeds the capacity of photosynthetic reaction centers to use energy absorbed by the light-harvesting antenna system. Thus, carotenoids are found wherever a chlorophyll is located, including the major photosynthetic components: PSI and PSII core complexes and Cytochrome b6/f complex. A further different carotenoid-dependent mechanism operates when carotenoids are located too far from Chlorophylls and excitation energy transfer is not allowed, consisting of suicide quenching of singlet oxygen. Indeed, b-carotene is consumed and its turnover is much faster [4] respect to that of xanthophylls, which are not destroyed by the photoprotective reactions they catalyze. Cells, however, can always find a function for carotenoids, including the apocarotenoids produced by their oxidation: b-cyclocitral is the major signal inducing gene expression and acclimation to excess light conditions [5]. Despite the detailed knowledge of carotenoid function one aspect of photoprotection is still unclear: how Chl biosynthesis can be regulated in order to avoid releasing Chl free in the membrane. The manuscript by Proctor and co-workers provide a logical answer to the question: the terminal step of chlorophyll biosynthesis, catalyzed by Chl G (Chlorophyll synthase), is activated by the xanthophyll zeaxanthin, which also stabilizes the interaction between, ChlG and the small dimeric Hlip proteins. This super-complex involves Ycf39, a component of the protein insertion system in the thylakoid membrane. The co-operation of these factors at the site where thylakoid polypeptide insertion and Chl biosynthesis occur offer an explanation for the tight regulation of Chl biosynthesis/assembly of the pigment-binding proteins: indeed, zeaxanthin-dependent association with HliD was found necessary for ChlG activity.

The way authors have traced this new function of xanthophyll is reverse genetic. Based on the observation that Synechocystis 6803 produces zeaxanthin while no know function makes explicitly use of this xanthophylls, they did produce a zeaxanthin-less strain. Previous work from members of the team had shown that TAG-ChlG formed a complex with HliD (a photoprotective protein quenching Chl fluorescence) and Ycf39 (an insertase carrying the precursor polypeptide for thylakoid insertion) in a pull-down assay. This complex was dissociated in the zeaxanthin-less strain but could be reconstituted by fusing zeaxanthin repleted membranes with the depleted ones. Re-association of the HliD and ChlD enhanced the catalyting activity of ChlD to release the accumulation of the ChlG substrate monovinyl (MV)-Chlide and resume Chl a biosynthesis. This is a brilliant experimental work. Authors discuss the glue-like activity of Zea on the basis of its rigid structure due to the extension of the conjugated double bond of the polyene to both b-ionone rings. This part of the work requires further research to clarify whether the complex-stabilizing function of Zea is a case of a general function for Zea in mediating protein–protein interactions or is specifically taylored for HliD and ChlD complex. The HliD–Ycf39 complex was maintained in the Zea-less mutant, supporting the latter hypothesis. Indeed, evidence from higher plants yielded several reports of changes in the aggregation state of proteic supercomplexes depending of the synthesis of Zea or structurally similar xanthophylls. To list a few examples, Zea synthesis in high light was needed for the dissociation of supercomplexes within the Photosystem II antenna system [6]. On the contrary, the lack of lutein caused dissociation of the major trimeric LHCII complex [7]. This work clearly demonstrates the role of zeaxanthin in the stabilization of protein–protein interaction(s) and co-ordinate apoprotein insertion in the membranes with the synthesis of the potentially harmful chromophore. Future work will elucidate the exact topological location of zeaxanthin within the complex and tell whether zeaxanthin is located at the interface between protein subunits or binds to allosteric sites to induce protein–protein interactions, via conformational change.

## Abbreviations

LHC: light-harvesting complex
ROS: reactive oxygen species
